# Supplementary material for: Human ribonuclease 1 serves as a secretory ligand of ephrin A4 receptor and induces breast tumor initiation
Source: Nat Commun. 2021 May 13;12:2788. doi: 10.1038/s41467-021-23075-2 (PMC8119676; doi:10.1038/s41467-021-23075-2)
Supplement: Supplementary file 1 — Supplementary information. [file 41467_2021_23075_MOESM1_ESM.pdf]

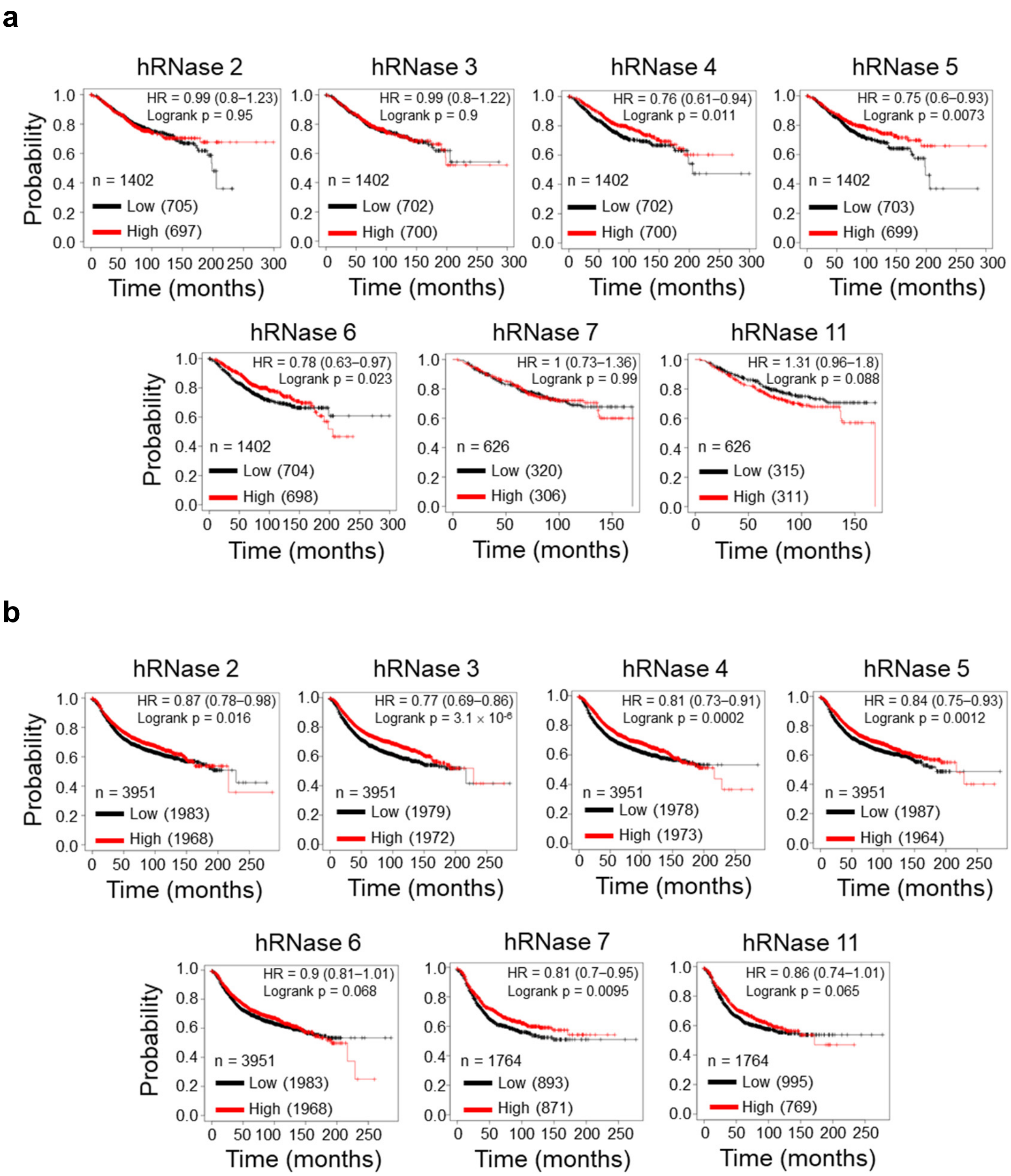

Supplementary Fig. 1. Survival curve analysis by screening the expression of hRNase family in human cancers. a and **b**, Prognostic correlation of survival analyses of breast cancer patients with high and low hRNases expression as indicated. Data was analyzed from the overall survival (**a**; OS) and the relapse free survival (**b**; RFS). Survival data were analyzed using Kaplan-Meier Plotter [Probe: 206111_at (hRNase 2); 206851_at (hRNase 3); 205158_at (hRNase 4); 205141_at

(hRNase 5); 213566_at (hRNase 6); 234699_at (hRNase 7); 231603_at (hRNase 11)]. HR, hazard ratio. All p values are calculated by Log-rank test.

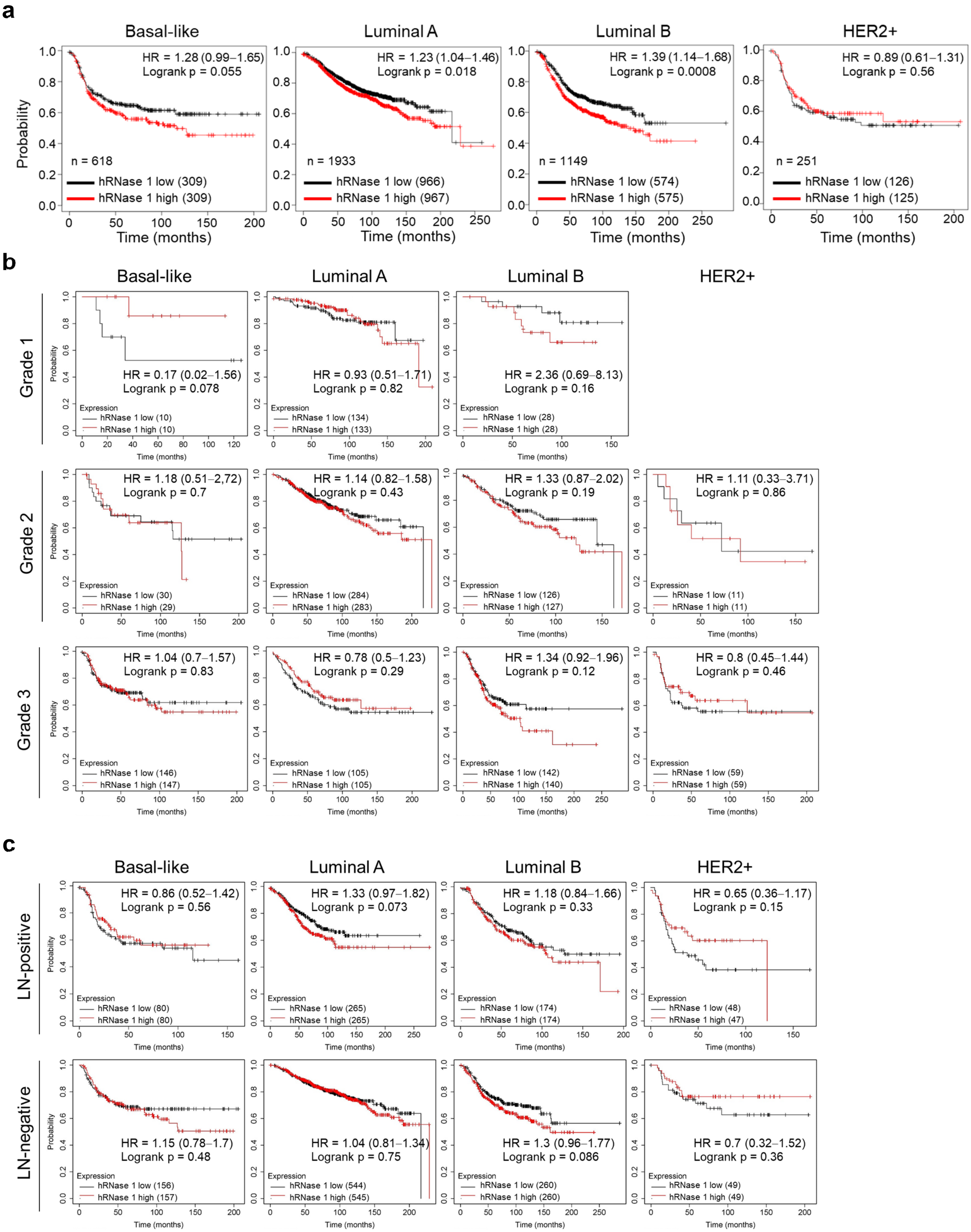
a
Basal-like
Luminal A
Luminal B
HER2+
Probability
Time (months)
HR = 1.28 (0.99–1.65)
Logrank p = 0.055
n = 618
hRNase 1 low (309)
hRNase 1 high (309)
HR = 1.23 (1.04–1.46)
Logrank p = 0.018
n = 1933
hRNase 1 low (966)
hRNase 1 high (967)
HR = 1.39 (1.14–1.68)
Logrank p = 0.0008
n = 1149
hRNase 1 low (574)
hRNase 1 high (575)
HR = 0.89 (0.61–1.31)
Logrank p = 0.56
n = 251
hRNase 1 low (126)
hRNase 1 high (125)
b
Basal-like
Luminal A
Luminal B
HER2+
Grade 1
HR = 0.17 (0.02–1.56)
Logrank p = 0.078
Expression
hRNase 1 low (10)
hRNase 1 high (10)
HR = 0.93 (0.51–1.71)
Logrank p = 0.82
hRNase 1 low (134)
hRNase 1 high (133)
HR = 2.36 (0.69–8.13)
Logrank p = 0.16
hRNase 1 low (28)
hRNase 1 high (28)
Grade 2
HR = 1.18 (0.51–2,72)
Logrank p = 0.7
hRNase 1 low (30)
hRNase 1 high (29)
HR = 1.14 (0.82–1.58)
Logrank p = 0.43
hRNase 1 low (284)
hRNase 1 high (283)
HR = 1.33 (0.87–2.02)
Logrank p = 0.19
hRNase 1 low (126)
hRNase 1 high (127)
HR = 1.11 (0.33–3.71)
Logrank p = 0.86
hRNase 1 low (11)
hRNase 1 high (11)
Grade 3
HR = 1.04 (0.7–1.57)
Logrank p = 0.83
hRNase 1 low (146)
hRNase 1 high (147)
HR = 0.78 (0.5–1.23)
Logrank p = 0.29
hRNase 1 low (105)
hRNase 1 high (105)
HR = 1.34 (0.92–1.96)
Logrank p = 0.12
hRNase 1 low (142)
hRNase 1 high (140)
HR = 0.8 (0.45–1.44)
Logrank p = 0.46
hRNase 1 low (59)
hRNase 1 high (59)
c
Basal-like
Luminal A
Luminal B
HER2+
LN-positive
HR = 0.86 (0.52–1.42)
Logrank p = 0.56
hRNase 1 low (80)
hRNase 1 high (80)
HR = 1.33 (0.97–1.82)
Logrank p = 0.073
hRNase 1 low (265)
hRNase 1 high (265)
HR = 1.18 (0.84–1.66)
Logrank p = 0.33
hRNase 1 low (174)
hRNase 1 high (174)
HR = 0.65 (0.36–1.17)
Logrank p = 0.15
hRNase 1 low (48)
hRNase 1 high (47)
LN-negative
HR = 1.15 (0.78–1.7)
Logrank p = 0.48
hRNase 1 low (156)
hRNase 1 high (157)
HR = 1.04 (0.81–1.34)
Logrank p = 0.75
hRNase 1 low (544)
hRNase 1 high (545)
HR = 1.3 (0.96–1.77)
Logrank p = 0.086
hRNase 1 low (260)
hRNase 1 high (260)
HR = 0.7 (0.32–1.52)
Logrank p = 0.36
hRNase 1 low (49)
hRNase 1 high (49)

Supplementary Fig. 2. High hRNase 1 expression correlates with poor patient survival in breast cancer patients with basal-like, luminal A, and luminal B subtypes. **a**, Prognostic correlation of the RFS of breast cancer patients in different breast cancer subtypes with different hRNase 1 levels as indicated. **b** and **c**, Prognostic correlation of the RFS of breast cancer patients with different grades (**b**) and lymph node (LN) statuses (**c**) in different breast cancer subtypes with different hRNase 1 levels as indicated. Data of grade 1 patients with of HER2+ is not provided in the database. All p values are calculated by Log-rank test (**a**–**c**).

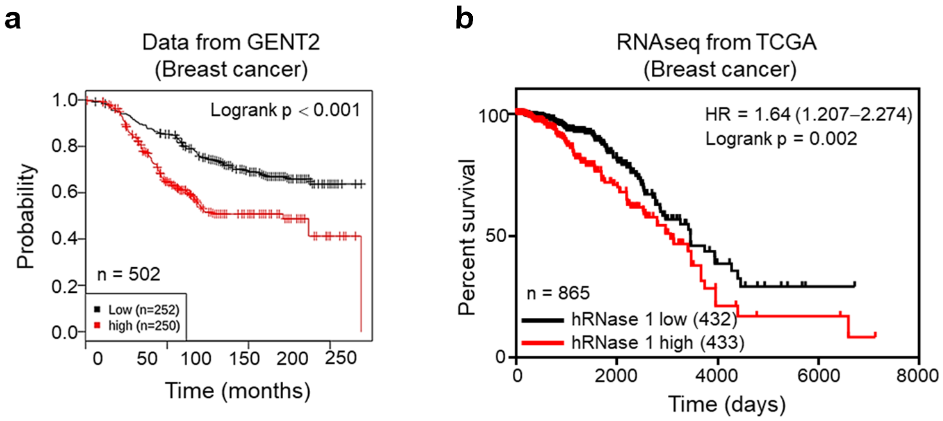

Supplementary Fig. 3. High hRNase 1 expression correlates with poor patient survival in two other independent databases of patients with breast cancer. a, Prognostic correlation of the OS of breast cancer patients with high and low hRNase 1 expression divided by the median value, analyzed the Kaplan-Meier Plotter from the GENT2 database (http://gent2.appex.kr/gent2/). **b**, Prognostic correlation of the OS of breast cancer patients with high and low hRNase 1 expression divided by the median value, analyzed the Kaplan-Meier survival curve from the UCSC Cancer Genome Browser (http://xena.ucsc.edu/welcome-to-ucsc-xena/) using the interpreted expression profile of TCGA breast invasive carcinoma by RNA sequencing (dataset ID: TCGA_BRCA_exp_HiSeqV2). Both p values are calculated by Log-rank test (**a**, **b**).

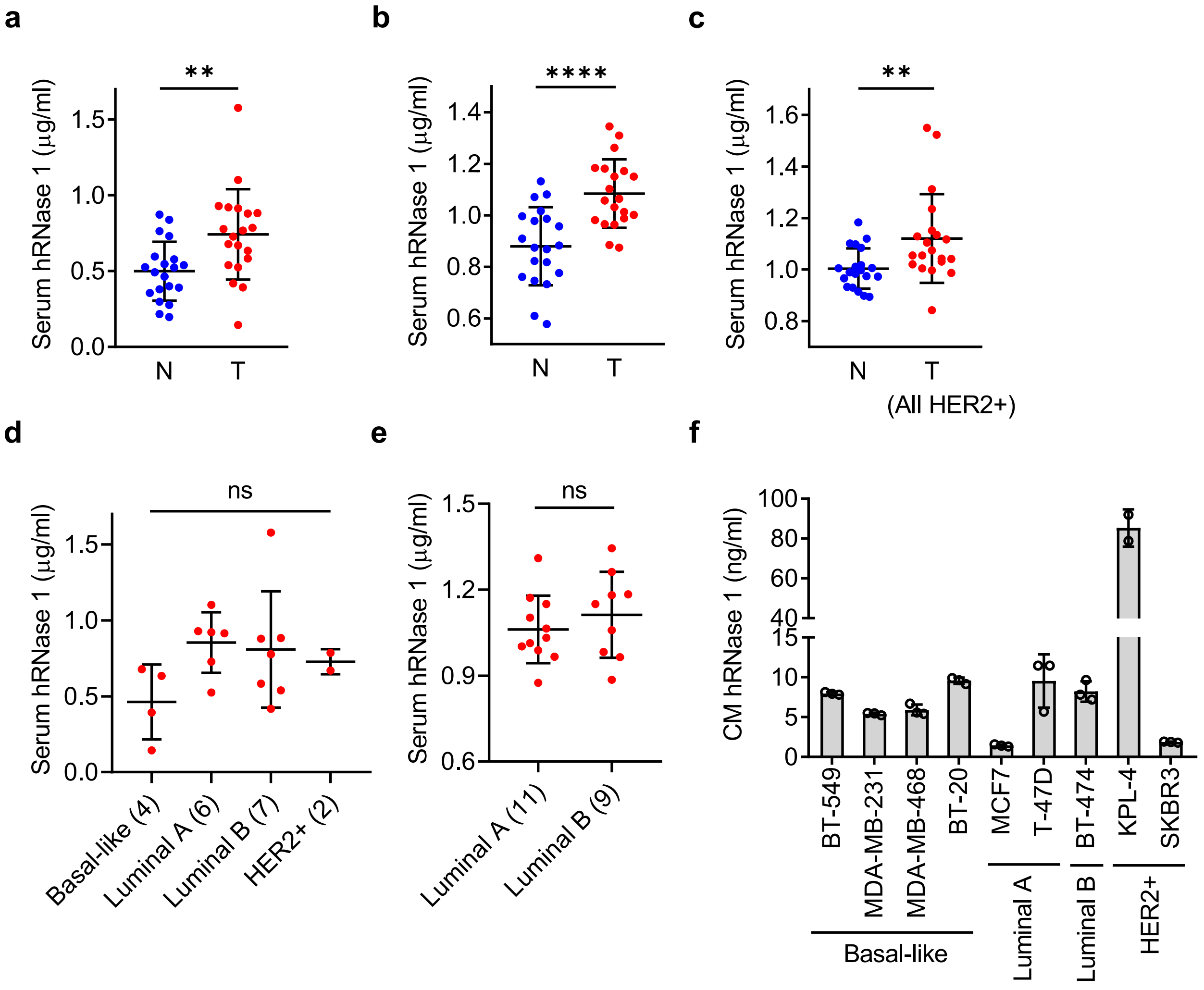

Supplementary Fig. 4. Serum samples of breast cancer patients show higher hRNase 1 expression than normal individuals. a–c, ELISA of hRNase 1 levels in three independent cohorts of serum samples of breast tumor patients (T; n = 20) compared with noncancerous individuals (N; n = 20). **p = 0.0041, ****p < 0.0001, **p = 0.0090, respectively. All patients in the cohort (**c**) belong to HER2+ subtype. **d**, ELISA of hRNase 1 levels in breast tumor serum samples from (**a**) subdivided into four molecular subtypes, including Basal-like (n = 4), Luminal A (n = 6), Luminal B (n = 7), and HER2+ (n = 2). **e**, ELISA of hRNase 1 levels in breast tumor serum samples from (**b**) subdivided into two molecular subtypes, including Luminal A (n = 11) and Luminal B (n

= 9). **f**, ELISA of hRNase 1 expression in the conditioned medium (CM) of breast cancer cells. Data represent three independent experiments. All error bars represent mean ± SD. $**p < 0.01$, $****p < 0.0001$, ns, not significant, two-tailed unpaired t test (**a**, **b**, **c**, **e**), One way ANOVA analysis (**d**). Source data are provided as a Source Data file.

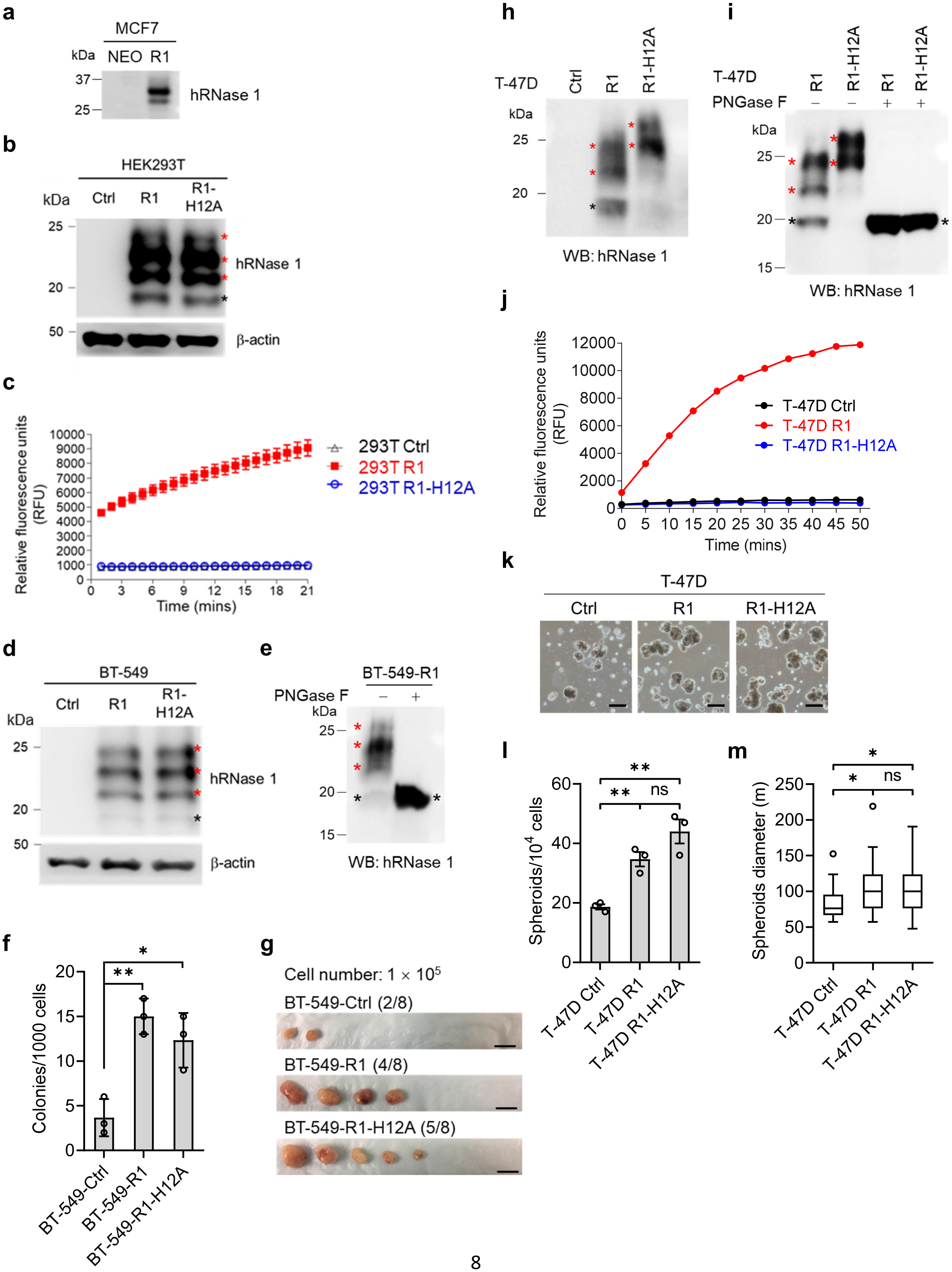
a
MCF7
kDa
NEO
R1
37
25
hRNase 1
b
HEK293T
kDa
Ctrl
R1
R1-H12A
25
20
50
hRNase 1
β-actin
c
Relative fluorescence units (RFU)
Time (mins)
293T Ctrl
293T R1
293T R1-H12A
d
BT-549
kDa
Ctrl
R1
R1-H12A
25
20
50
hRNase 1
β-actin
e
BT-549-R1
PNGase F
kDa
25
20
15
WB: hRNase 1
f
Colonies/1000 cells
BT-549-Ctrl
BT-549-R1
BT-549-R1-H12A
g
Cell number: 1 × 10^5
BT-549-Ctrl (2/8)
BT-549-R1 (4/8)
BT-549-R1-H12A (5/8)
h
T-47D
Ctrl
R1
R1-H12A
kDa
25
20
WB: hRNase 1
i
T-47D
PNGase F
R1
R1-H12A
kDa
25
20
15
WB: hRNase 1
j
Relative fluorescence units (RFU)
Time (mins)
T-47D Ctrl
T-47D R1
T-47D R1-H12A
k
T-47D
Ctrl
R1
R1-H12A
l
Spheroids/10^4 cells
T-47D Ctrl
T-47D R1
T-47D R1-H12A
ns
m
Spheroids diameter (m)
T-47D Ctrl
T-47D R1
T-47D R1-H12A
ns

Supplementary Fig. 5. hRNase 1 increases the sphere-forming ability and enhances the tumor-initiating capability of breast cancer cells independently of its ribonucleolytic activity. **a**, WB of secreted proteins from CM in MCF7 control cells (MCF7-NEO) and hRNase 1 expressing MCF7 cells (MCF7-R1) with hRNase 1 antibody. **b**, WB of cell lysates in Flag-tagged hRNase 1 in HEK293T transfected with an empty control vector (Ctrl), R1, and R1-H12A with Flag and β-actin antibodies. **c**, Analysis of RNase enzymatic activity by an RNaseAlert® Lab Test kit in CM collected from HEK293T transfected with the indicated plasmids. Data are representative of two independent experiments in triplicate. Error bars represent mean ± SD. **d**, WB of cell lysates in BT-549 stable transfectants expressing empty control (Ctrl), R1, and R1-H12A with Flag and β-actin antibodies. **e**, WB of secreted proteins from CM in BT-549-R1 treated without or with PNGase F. **f**, Quantification of soft agar colony formation assay of cells from (**d**). Ctrl vs R1, **p = 0.002, Ctrl vs R1-H12A, *p = 0.015. **g**. Representative images of limiting dilution assay of the indicated BT-549 stable clones (1×10^5) from Figure 2g. The number of tumor-forming mice within each group is shown in the parentheses. Bar, 1 cm. **h**, WB of secreted proteins from CM in T-47D stable transfectants expressing empty control (Ctrl), R1, and R1-H12A with hRNase 1 antibody. **i**, WB of secreted proteins from CM in T-47D R1 and T-47D R1-H12A treated without or with PNGase F. **j**, Analysis of RNase enzymatic activity by an RNaseAlert® Lab Test kit in CM collected from (**h**). **k**, Representative images of primary spheres from (**h**). Bar, 100 μm. **l** and **m**, Quantification of spheroid number (**l**) and diameter (**m**) of spheroid formation assay from (**h**). **l**, Ctrl vs R1, **p = 0.0033, Ctrl vs R1-H12A, **p = 0.0036. **m**, Ctrl vs R1, *p = 0.0296, Ctrl vs R1-H12A, *p = 0.0497. Box plots, including T-47D Ctrl (n = 23), T-47D R1 (n = 30), and T-47D R1-H12A (n = 36), indicate minima (lower end of whisker), maxima (upper end of whisker), median (centre), 25th percentile (bottom of box) and 75th percentile (top of box) as well as outliers

(single points) (**m**). Error bars represent mean ± SD (**f**) and mean ± SEM (**l**), n = 3 independent experiments (**f**, **l**). *p < 0.05, **p < 0.01, ns, not significant, two-tailed unpaired t test (**f**, **l**, **m**). Each experiment was repeated a second time with similar results (**a**, **b**, **d**, **e**, **h**, **i**). Antibodies used in WB, hRNase 1 (Sigma-Aldrich, #HPA001140); Flag (Sigma-Aldrich, #F3165); β-actin (Sigma-Aldrich, #A2228). Red asterisk, glycosylated hRNase 1; black asterisk, non-glycosylated hRNase 1. Source data are provided as a Source Data file.

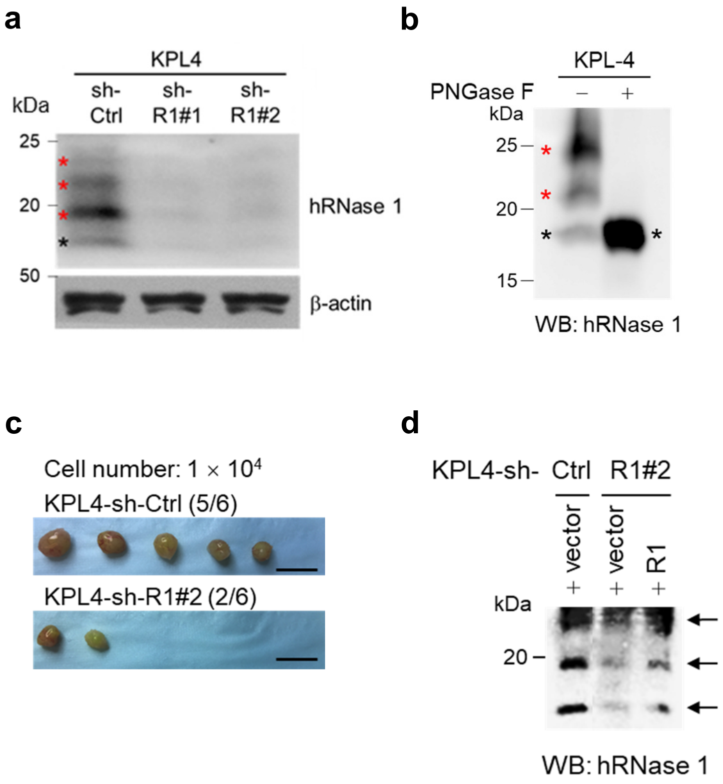

Supplementary Fig. 6. Silencing hRNase 1 decreases the tumor-initiating capability in KPL4 cells. a, WB of cell lysates in KPL4 stable transfectants knocking down hRNase 1 and empty control with hRNase 1 and β-actin antibodies. **b**, WB of secreted proteins from CM in KPL4 treated without or with PNGase F. **c**. Representative images of limiting dilution assay of the indicated KPL4 stable clones (1×10^4) from Figure 2j. The number of tumor-forming mice within each group is shown in the parentheses. Bar, 1 cm. **d**, WB of secreted proteins from CM in the reconstitution of vector control or hRNase 1 in hRNase 1-knockdown KPL4 (KPL4-sh-R1#2) and control cells (KPL4-sh-Ctrl). Each experiment was repeated a second time with similar results (**a**, **b**, **d**). Antibodies used in WB, hRNase 1 (Sigma-Aldrich, #HPA001140); β-actin (Sigma-Aldrich, #A2228). Red asterisk, glycosylated hRNase 1; black asterisk, non-glycosylated hRNase 1. Source data are provided as a Source Data file.

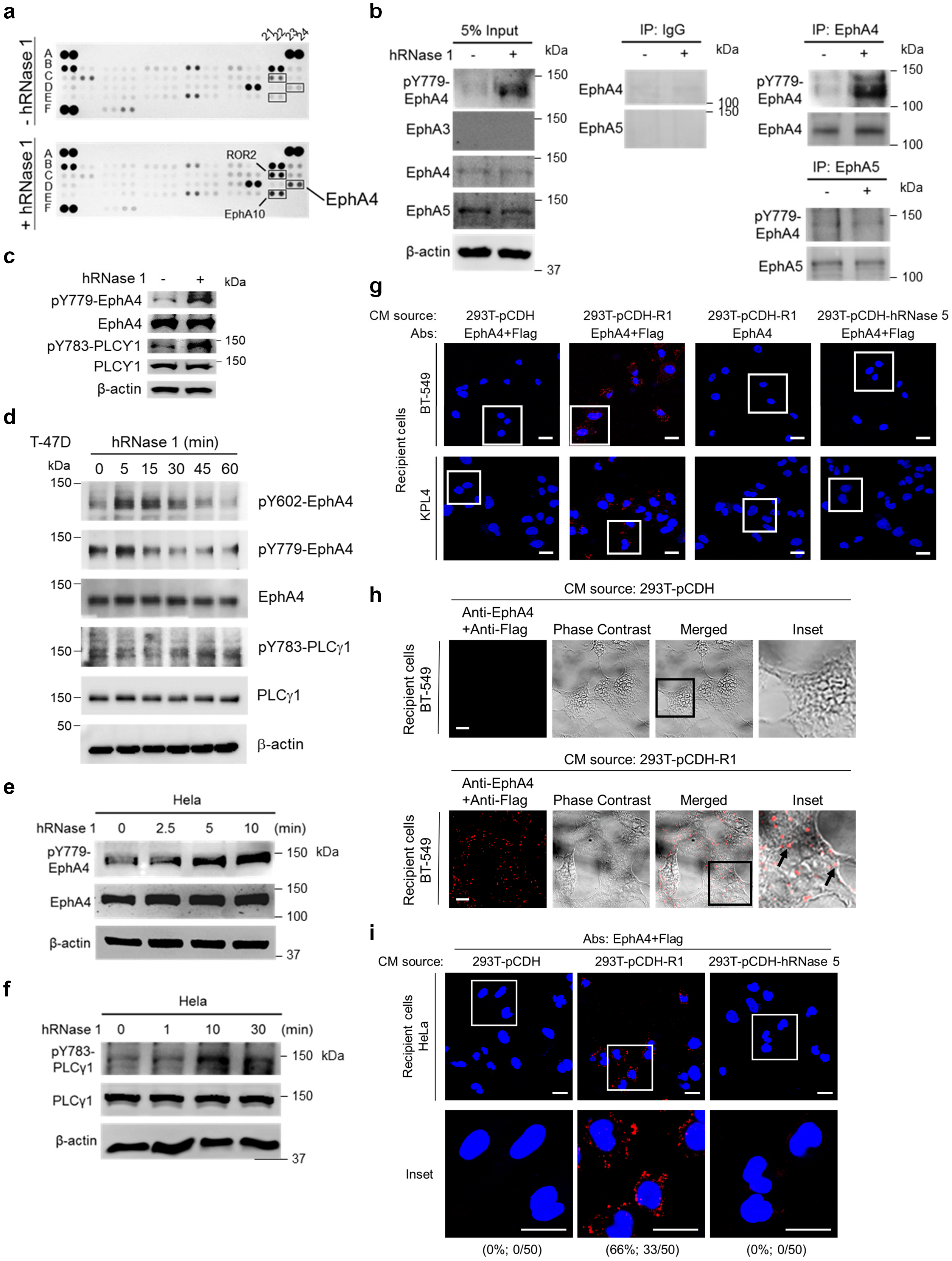

a
- hRNase 1
+ hRNase 1
A
B
C
D
E
F
21
22
23
24
ROR2
EphA4
EphA10
b
5% Input
IP: IgG
IP: EphA4
IP: EphA5
hRNase 1
-
+
kDa
pY779-EphA4
EphA3
EphA4
EphA5
β-actin
150
100
37
c
hRNase 1 - +
kDa
pY779-EphA4
EphA4
pY783-PLCY1
PLCY1
β-actin
150
d
T-47D
hRNase 1 (min)
kDa
0 5 15 30 45 60
pY602-EphA4
pY779-EphA4
EphA4
pY783-PLCγ1
PLCγ1
β-actin
150
50
e
Hela
hRNase 1 0 2.5 5 10 (min)
pY779-EphA4
EphA4
β-actin
150 kDa
100
37
f
Hela
hRNase 1 0 1 10 30 (min)
pY783-PLCγ1
PLCγ1
β-actin
150 kDa
37
g
CM source:
Abs:
293T-pCDH
EphA4+Flag
293T-pCDH-R1
EphA4+Flag
293T-pCDH-R1
EphA4
293T-pCDH-hRNase 5
EphA4+Flag
Recipient cells
BT-549
KPL4
h
CM source: 293T-pCDH
CM source: 293T-pCDH-R1
Anti-EphA4
+Anti-Flag
Phase Contrast
Merged
Inset
Recipient cells
BT-549
i
Abs: EphA4+Flag
CM source:
293T-pCDH
293T-pCDH-R1
293T-pCDH-hRNase 5
Recipient cells
HeLa
Inset
(0%; 0/50)
(66%; 33/50)
(0%; 0/50)

Supplementary Fig. 7. hRNase 1 stimulates EphA4 signaling and interacts with EphA4 in several cancer cell lines. a, Human phospho-RTK antibody array analysis of BT-549 cells treated with or without recombinant hRNase 1 protein purified from HEK293 cells (1 μg/ml) for 30 min after serum starvation for 3 hr. Three pairs of positive signals in duplicate coordinates (- hRNase 1 comparing to + hRNase 1) are shown in EphA4 (D23/D24), EphA10 (E21/E22), and ROR2 (C21/C22). **b**, Immunoprecipitation (IP) of BT-549 cells treated with or without 1 μg/ml hRNase 1 for 30 min, followed by WB with the indicated antibodies. Immunoglobulin G (IgG) was used as a control for IP. **c**, WB of BT-549 cells treated with or without hRNase 1 for 30 min with the indicated antibodies. **d**, WB of T-47D cells treated with or without hRNase 1 at various time points with the indicated antibodies. **e** and **f**, WB of pY779-EphA4 and EphA4 (**e**) as well as pY783-PLCγ1 and PLCγ1 (**f**) in HeLa cells treated with hRNase 1 (1 μg/ml) at different time points as indicated. **g**, Duolink *in situ* proximity ligation assay (PLA) of BT-549 and KPL4 cells, treated with CM collected from 293T-pCDH, 293T-pCDH-R1, or 293T-pCDH-hRNase 5 for 30 min, and stained with EphA4 only or EphA4 plus Flag antibodies as indicated. Insets for Figure 3f, BT-549, enlargements of the boxed areas at 9× magnification; KPL4, enlargements of the boxed areas at 12.25× magnification. Bar, 25 μm. **h**, Duolink *in situ* PLA of BT-549 cells, treated with CM collected from 293T-pCDH or 293T-pCDH-R1 for 30 min, and stained with EphA4 and Flag antibodies. Arrows in the insets, red dots, positive Duolink *in situ* signals. Insets, enlargements of the boxed areas (6× magnification). Bar, 10 mm. **i**, Duolink *in situ* PLA of HeLa cells, treated with CM collected from 293T-pCDH, 293T-pCDH-R1, or 293T-pCDH-hRNase 5 for 30 min, and stained with EphA4 and Flag antibodies. Inset, 9× magnification. Bar, 25 μm. The quantified results in the parentheses showing the percentage of cells with positive PLA signals calculated

from a pool of 50 cells. Each experiment was repeated an additional time with similar results (**a–i**).

Source data are provided as a Source Data file.

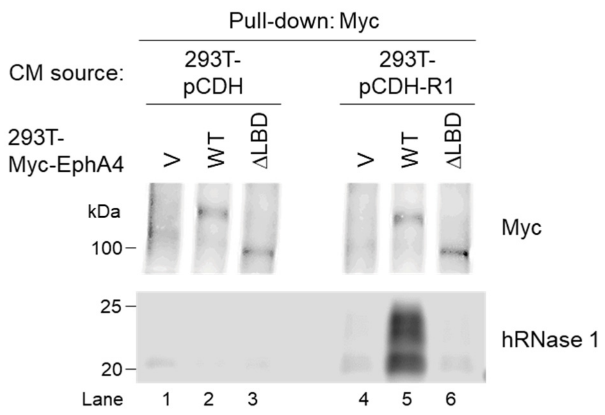

Supplementary Fig. 8. The extracellular ligand-binding domain of EphA4 is critical for hRNase 1 binding. *In vitro* binding assay of 293T stable transfectants with empty vector (V), Myc-tagged EphA4 containing wild-type (WT), and ligand-binding domain deletion (ΔLBD), incubated with CM collected from 293T stable transfectant expressing empty vector (293T-pCDH) or Flag-tagged hRNase 1 (293T-pCDH-R1). Data are representative of two independent experiments with similar results. Source data are provided as a Source Data file.

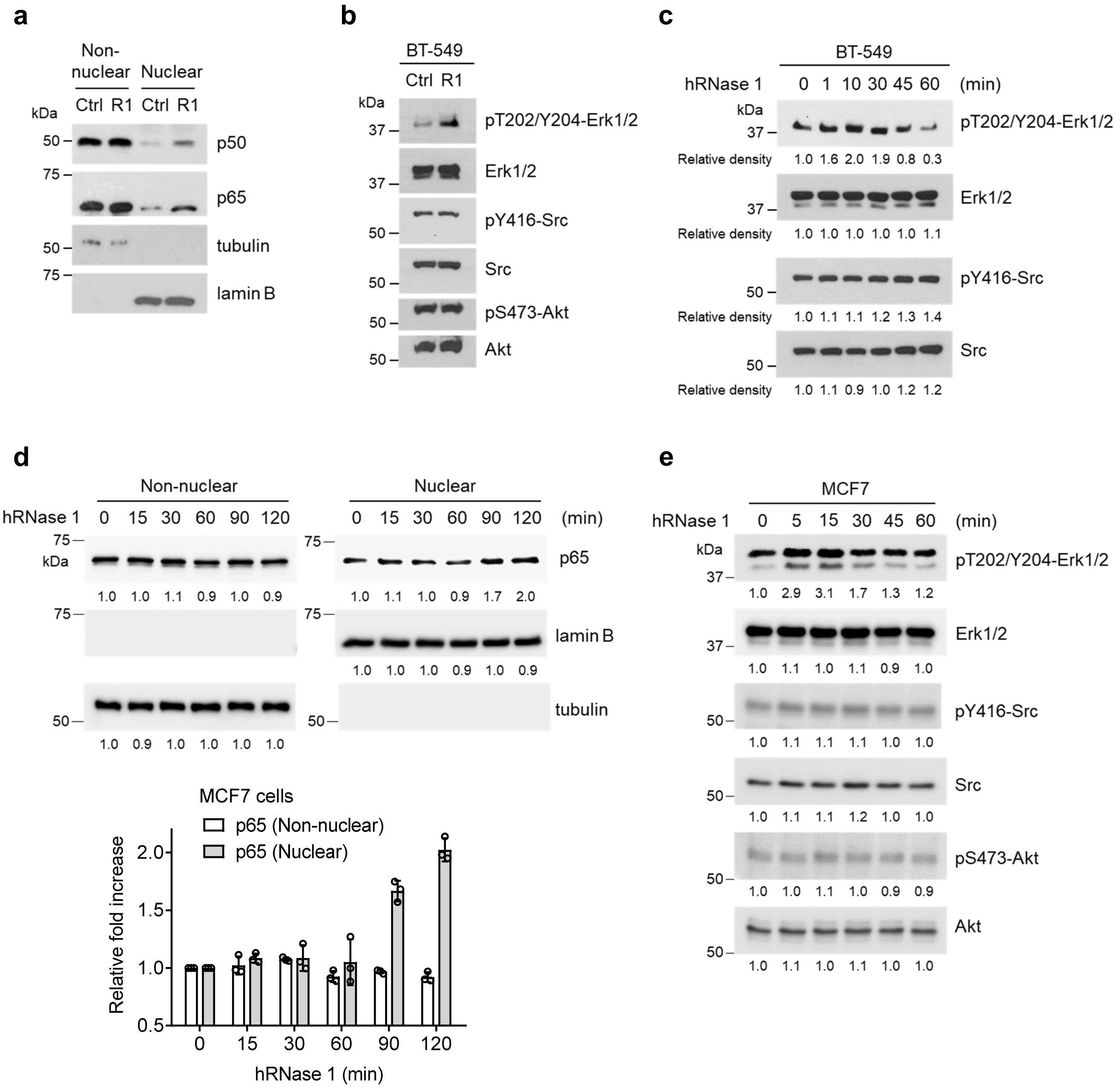

Supplementary Fig. 9. hRNase 1 activates signaling pathways of NF-κB and Erk, but not Src or Akt, in BT-549 and MCF7 cells. **a**, Cellular fractionation of BT-549-Ctrl and BT-549-R1 stable clones, followed by WB with the indicated antibodies. Tubulin and lamin B were used as markers for the non-nuclear and nuclear portions, respectively. **b**, WB of BT-549-Ctrl and BT-549-R1 stable clones with the indicated antibodies. **c**, WB of BT-549 cells treated with

recombinant hRNase 1 protein purified from HEK293 cells (1 μg/ml) at various time points with the indicated antibodies. **d**, Top, cellular fractionation of MCF7 cells treated with hRNase 1 (1 μg/ml) at various time points, followed by WB with the indicated antibodies. Bottom, quantification of fold increase normalized against that at time 0 min. Error bars represent mean ± SD, n = 2 independent experiments. **e**, WB of MCF7 cells treated with hRNase 1 (1 μg/ml) at various time points with the indicated antibodies. Relative density of signals were quantified using ImageJ. Data are representative of two (**a**) or three (**b**, **c**, **e**) independent experiments with similar results. Source data are provided as a Source Data file.

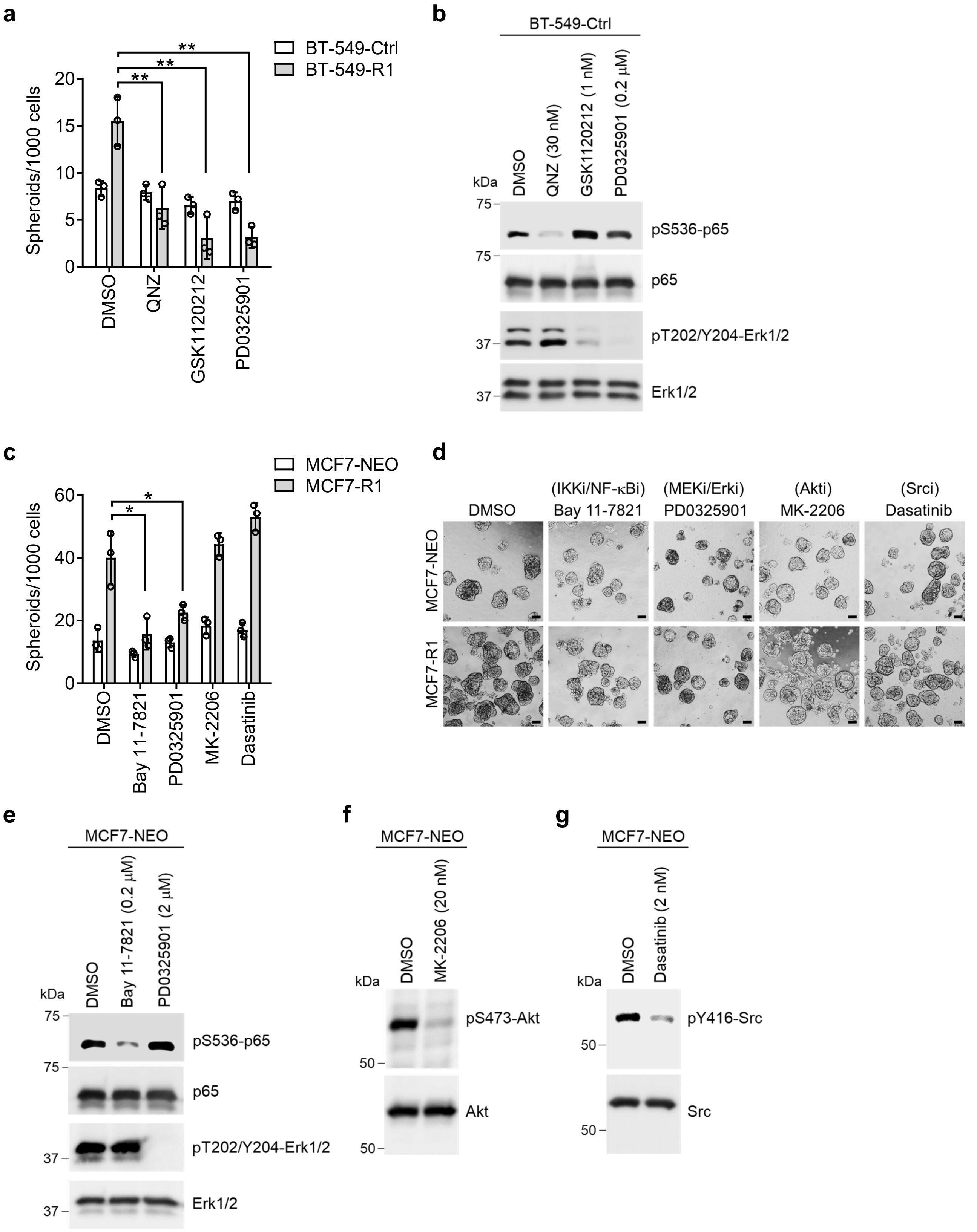
a
BT-549-Ctrl
BT-549-R1
Spheroids/1000 cells
0
5
10
15
20
**
**
**
DMSO
QNZ
GSK1120212
PD0325901
b
BT-549-Ctrl
kDa
DMSO
QNZ (30 nM)
GSK1120212 (1 nM)
PD0325901 (0.2 μM)
75
pS536-p65
75
p65
37
pT202/Y204-Erk1/2
37
Erk1/2
c
MCF7-NEO
MCF7-R1
Spheroids/1000 cells
0
20
40
60
*
*
DMSO
Bay 11-7821
PD0325901
MK-2206
Dasatinib
d
DMSO
(IKKi/NF-κBi)
Bay 11-7821
(MEKi/Erki)
PD0325901
(Akti)
MK-2206
(Srci)
Dasatinib
MCF7-NEO
MCF7-R1
e
MCF7-NEO
kDa
DMSO
Bay 11-7821 (0.2 μM)
PD0325901 (2 μM)
75
pS536-p65
75
p65
37
pT202/Y204-Erk1/2
37
Erk1/2
f
MCF7-NEO
kDa
DMSO
MK-2206 (20 nM)
pS473-Akt
50
Akt
50
g
MCF7-NEO
kDa
DMSO
Dasatinib (2 nM)
pY416-Src
50
Src
50

Supplementary Fig. 10. hRNase 1 induces spheroid formation via the MEK/Erk and IKK/NF-κB activation pathways in BT-549 and MCF7 cells. a, Quantification of spheroid formation assay in BT-549-Ctrl and BT-549-R1 stable clones incubated with the inhibitors against IKK/NF-κB (QNZ, 30 nM) and MEK/Erk (GSK1120212, 1 nM; PD0325901, 0.2 μM). DMSO vs QNZ, **p = 0.0097, DMSO vs GSK1120212, **p = 0.0032, DMSO vs PD0325901, p = **0.0016. **b**, WB of BT-549-Ctrl cells treated with DMSO or various inhibitors for 1 day with the indicated antibodies. **c**, Quantification of spheroid formation assay in MCF7-NEO and MCF7-R1 stable clones incubated with the inhibitors against IKK/NF-κB (Bay 11-7821, 0.2 μM), MEK/Erk (PD0325901, 2 nM), Akt (MK-2206, 20 nM), and Src (Dasatinib, 2 nM). DMSO vs Bay 11-7821, *p = 0.0133, DMSO vs PD0325901, p = *0.0275. **d**, Representative images of sphere formation assay from (**c**). Bar, 100 μm. **e–g**, WB of MCF7-NEO cells treated with DMSO or various inhibitors for 1 day with the indicated antibodies. Error bars represent mean ± SD. Data are representative of two (**b**, **e–g**) or three (**a**, **c**, **d**) independent experiments. *p < 0.05, **p < 0.01, two-tailed unpaired t test. Source data are provided as a Source Data file.

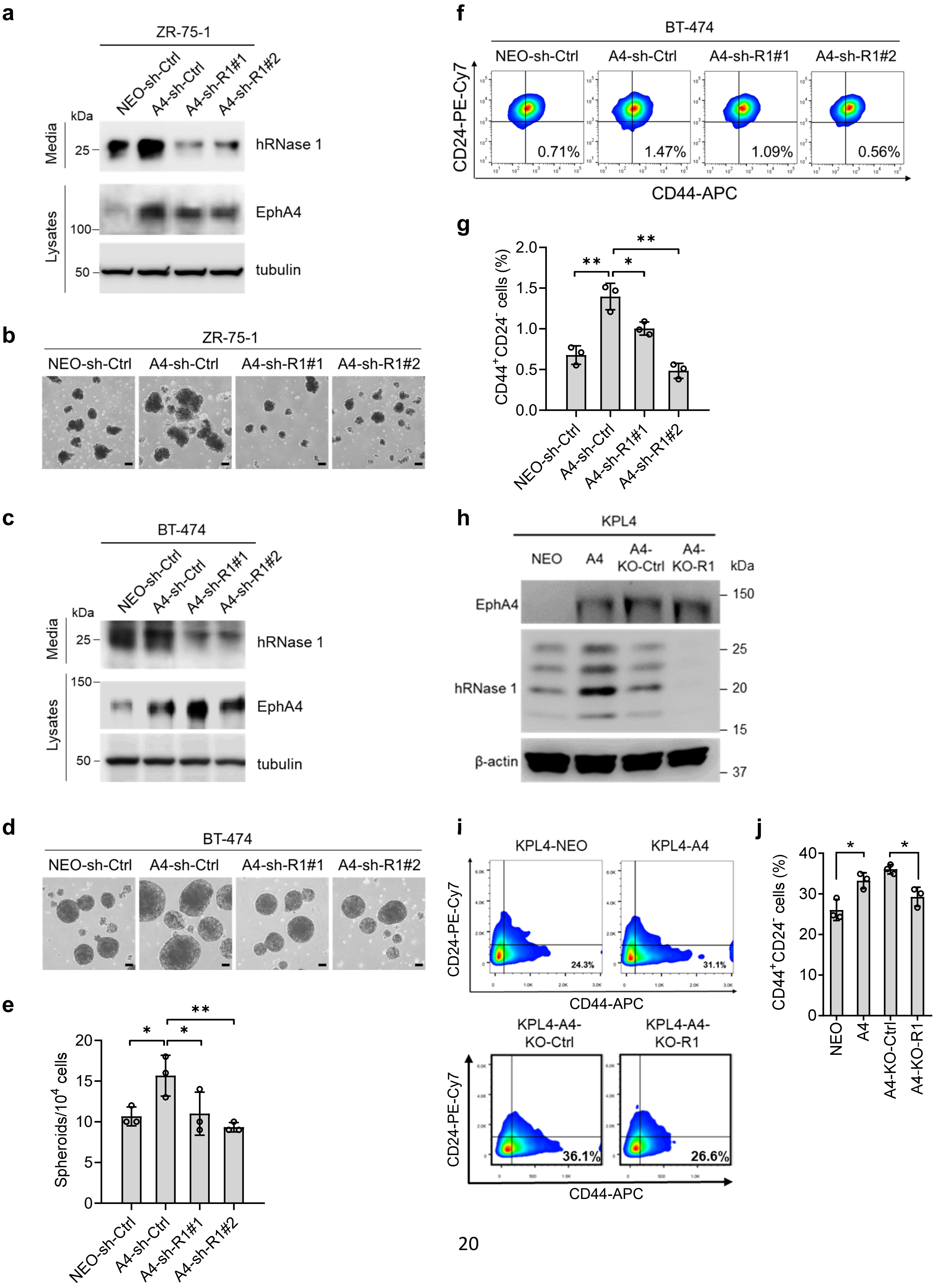
a
ZR-75-1
NEO-sh-Ctrl
A4-sh-Ctrl
A4-sh-R1#1
A4-sh-R1#2
kDa
Media
Lysates
hRNase 1
EphA4
tubulin
b
c
BT-474
d
e
Spheroids/10^4 cells
f
CD24-PE-Cy7
CD44-APC
0.71%
1.47%
1.09%
0.56%
g
CD44+CD24- cells (%)
h
KPL4
NEO
A4
A4-KO-Ctrl
A4-KO-R1
β-actin
i
KPL4-NEO
KPL4-A4
KPL4-A4-KO-Ctrl
KPL4-A4-KO-R1
24.3%
31.1%
36.1%
26.6%
j

Supplementary Fig. 11. Silencing hRNase 1 reduces EphA4-promoted spheroid formation and $CD44^{+}CD24^{-}$ cell population. **a**, WB of ZR-75-1 control cells (NEO-sh-Ctrl), ectopic EphA4 expressing ZR-75-1 cells (A4-sh-Ctrl), and ZR-75-1-A4 knockdown hRNase 1 cells (A4-sh-R1#1 and A4-sh-R1#2) blotted with EphA4 and hRNase 1 antibodies. **b**, Representative images of primary spheres of the indicated ZR-75-1 stable clones from (**a**). Bar, 100 μm. **c**, WB of BT-474 control cells (NEO-sh-Ctrl), ectopic EphA4 expressing BT-474 cells (A4-sh-Ctrl), and BT-474-A4 knockdown hRNase 1 cells (A4-sh-R1#1 and A4-sh-R1#2) blotted with EphA4 and hRNase 1 antibodies. **d**, Representative images of primary spheres of the indicated BT-474 stable clones from (**c**). Bar, 100 μm. **e**, Quantification of spheroid formation assay of the indicated BT-474 stable clones. One-tailed p values are shown. NEO-sh-Ctrl vs A4-sh-Ctrl, *p = 0.0176, A4-sh-Ctrl vs A4-sh-R1#1, *p = 0.0456, A4-sh-Ctrl vs A4-sh-R1#2, **p = 0.0066. **f**, Flow cytometric analysis of membrane CD44 and CD24 expression of the indicated BT-474 stable clones. **g**, Quantification of flow cytometric analysis from (**f**). NEO-sh-Ctrl vs A4-sh-Ctrl, **p = 0.0033, A4-sh-Ctrl vs A4-sh-R1#1, *p = 0.0200, A4-sh-Ctrl vs A4-sh-R1#2, **p = 0.0011. **h**, WB of EphA4 and hRNase 1 in KPL4 control cells (KPL4-NEO), ectopic EphA4 expressing KPL4 cells (KPL4-A4), and KPL4-A4 knockout hRNase 1 (KO-R1) or control (KO-Ctrl) cells. **i**, Flow cytometric analysis of membrane CD44 and CD24 expression in KPL4 cells as indicated. The percentages of $CD44^{+}CD24^{-}$ cells are shown in the lower right quadrant of each panel. **j**, Quantification of flow cytometric analysis from (**i**). NEO vs A4, *p = 0.0182, A4-KO-Ctrl vs A4-KO-R1, *p = 0.0110. All error bars represent mean ± SD. Data represent two (**a**, **c**, **h**) or three (**b**, **d**, **e**, **g**, **j**) independent experiments. *p < 0.05, **p < 0.01, unpaired t test, one-tailed (**e**) and two-tailed (**g**, **j**). Source data are provided as a Source Data file.

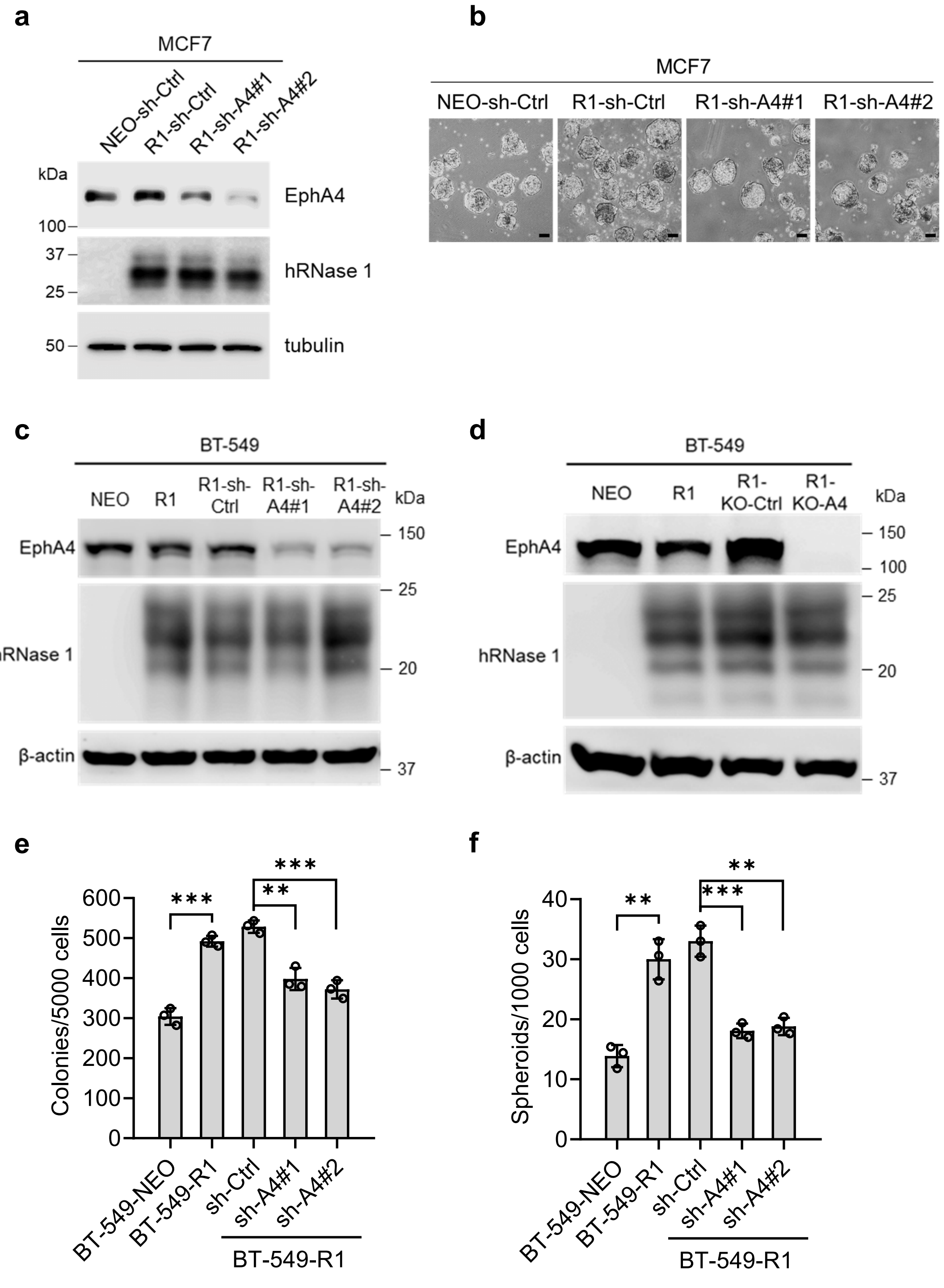

Supplementary Fig. 12. Silencing EphA4 reduces hRNase 1-mediated spheroid formation. a, WB of MCF7 control cells (NEO-sh-Ctrl), hRNase 1 expressing MCF7 cells (R1-sh-Ctrl), and MCF7-R1 knockdown EphA4 cells (R1-sh-A4#1 and R1-sh-A4#2) blotted with EphA4 and

hRNase 1 antibodies. **b**, Representative images of primary spheres of the indicated MCF7 stable clones from (**a**). Bar, 100 µm. **c**, WB of EphA4 and hRNase 1 in BT-549 control cells (BT-549-NEO), hRNase 1 expressing BT-549 cells (BT-549-R1), and BT-549-R1 transfected with shRNA of two different sequences against EphA4 (sh-A4#1 and sh-A4#2) or with scrambled control (sh-Ctrl). **d**, WB of EphA4 and hRNase 1 in BT-549-NEO, BT-549-R1, and BT-549-R1 knockout EphA4 (KO-A4) or control (KO-Ctrl) cells. **e**, Quantification of soft agar colony formation assay of the indicated BT-549 stable clones from (**c**). NEO vs R1, ***p = 0.0002, R1-sh-Ctrl vs R1-sh-A4#1, **p = 0.0021, R1-sh-Ctrl vs R1-sh-A4#2, ***p = 0.0006. **f**, Quantification of spheroid formation assay of the indicated BT-549 stable clones from (**c**). NEO vs R1, **p = 0.0019, R1-sh-Ctrl vs R1-sh-A4#1, ***p = 0.0008, R1-sh-Ctrl vs R1-sh-A4#2, **p = 0.0012. The experiments were repeated once (**a**, **c**, **d**) and twice (**b**) with similar results. Data are representative of three independent experiments in triplicate (**e**, **f**). All error bars represent mean ± SD. **p < 0.01, ***p < 0.001, two-tailed unpaired t test. Source data are provided as a Source Data file.

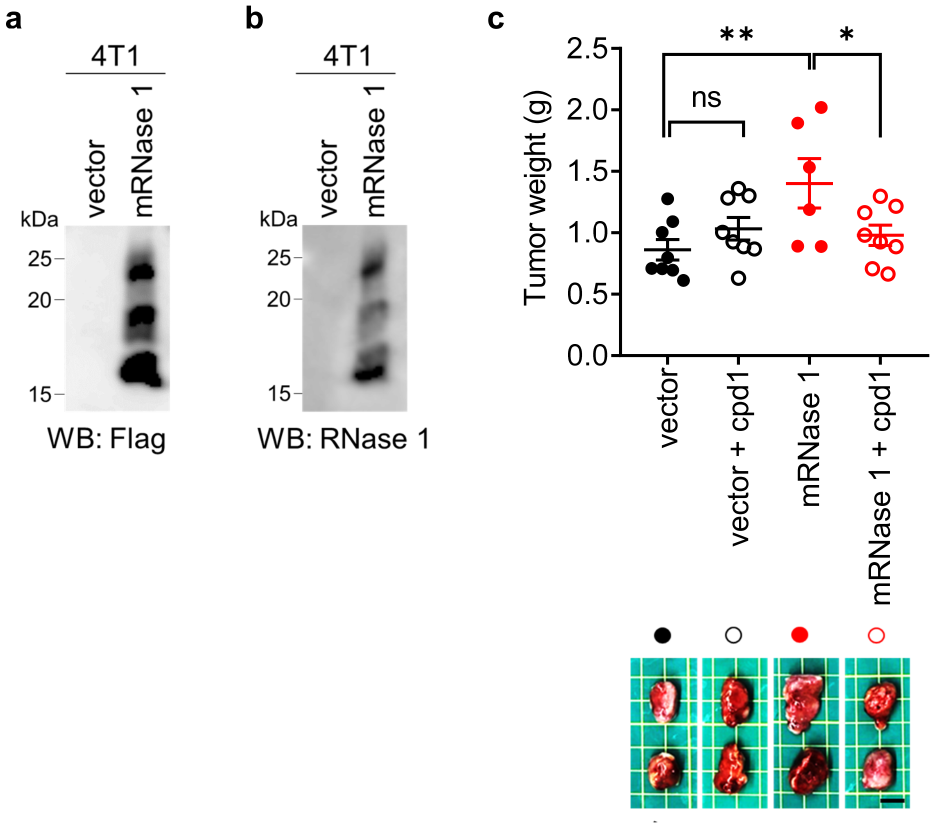

Supplementary Fig. 13. RNase 1-EphA4 axis contributes to breast tumorigenesis. a and **b**, WB analysis of mouse 4T1 mammary tumor cells expressing mouse RNase 1 (mRNase 1) and vector control blotted with antibodies against Flag (**a**) or RNase 1 (**b**; Santa Cruz Biotechnology, #sc-169198). The experiments were repeated an additional time with similar results. **c**, Tumor weight analysis (top) and representative images (bottom) of mice subcutaneously injected with the indicated 4T1 cells in (**a**) followed by treatment with solvent or compound 1 (cpd1). Bar, 1 cm. n = 8 per group except for the mRNase 1 group (n = 6). Vector vs mRNase 1, **p = 0.0089, mRNase 1 vs mRNase 1 + cpd1, *p = 0.0264. Error bars represent mean ± SEM. *p < 0.05, **p < 0.01, ns, not significant, one-tailed unpaired t test. Source data are provided as a Source Data file.

a

		phospho-EphA4		
		Low	High	Total
hRNase 1	Low	16	8	24
	High	4	20	24
	Total	20	28	48

p = 0.0001

b

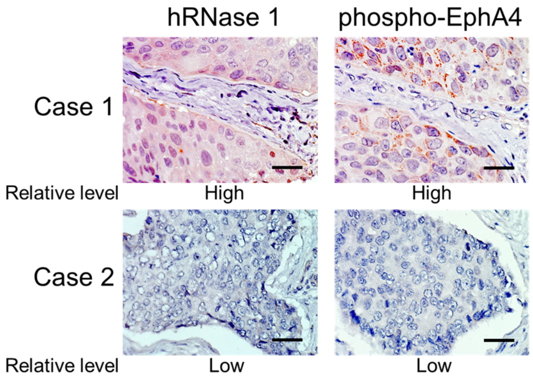

Supplementary Fig. 14. Pathological relevance between hRNase 1 expression and EphA4 activation in breast cancer. a, Quantification of IHC staining for the correlation between hRNase 1 and phospho-EphA4-Y779 using human breast tumor tissue microarray analysis (Pantomics Inc., #BRC961; Pearson Chi-Square test). **b**, Two representative cases of IHC staining from (**a**). The experiment was repeated a second time with similar results. Bar, 50 μm.

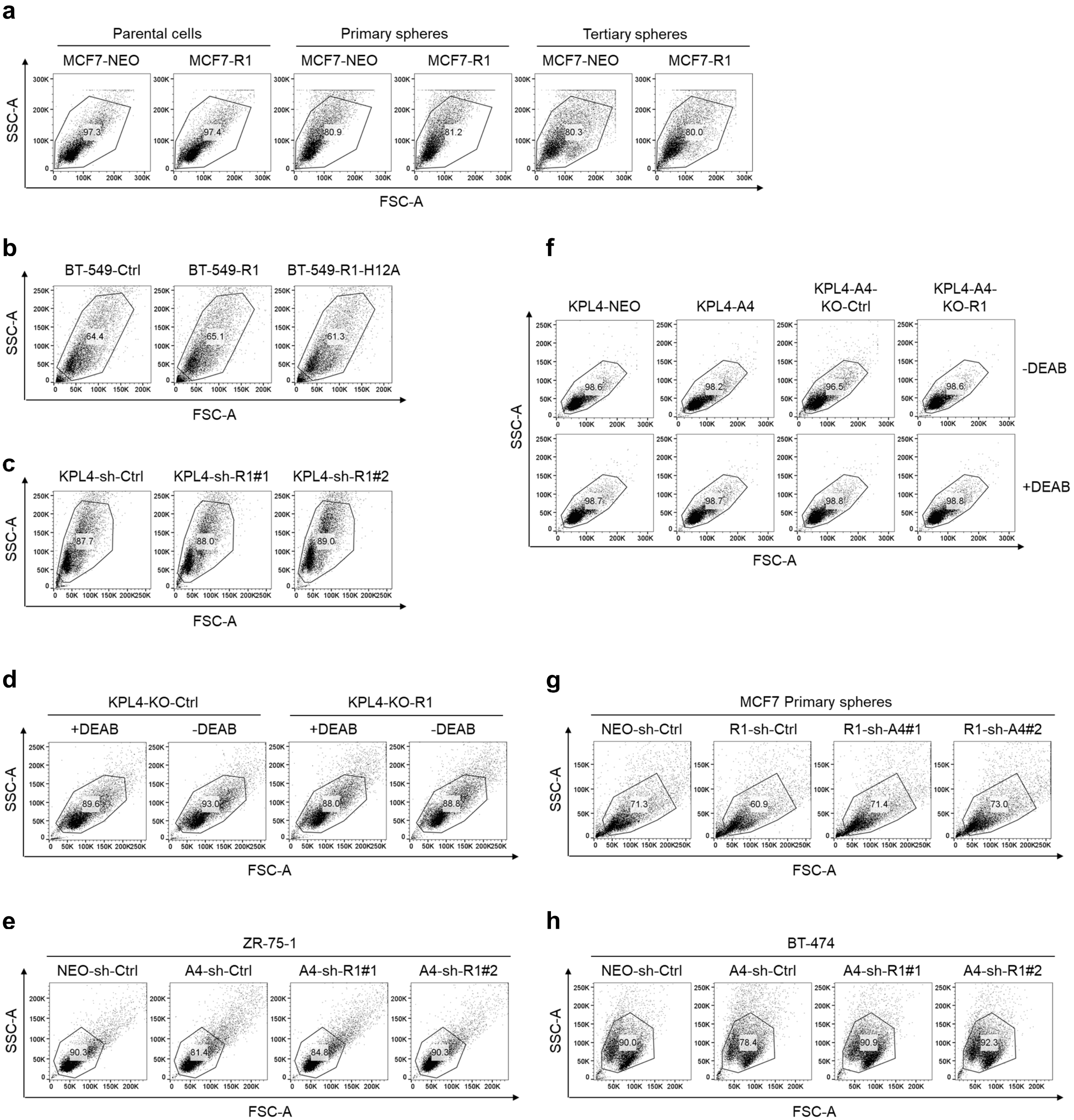

Supplementary Fig. 15. Gating strategies based on FSC-A/SSC-A parameters used for flow cytometric analysis of CD24/CD44 expression and ALDEFLUOR assay. a, Gating strategy to

determine the percentage of $CD24^{-}/CD44^{+}$ cells in the indicated MCF7 stable clones (parental cells) and primary and tertiary spheres derived from the indicated MCF7 stable clones presented on Fig. 2c. **b**, Gating strategy to determine the percentage of $CD24^{-}/CD44^{+}$ cells in BT-549 stable clones presented on Fig. 2f. **c**, Gating strategy to determine the percentage of $CD24^{-}/CD44^{+}$ cells in KPL4 stable clones presented on Fig. 2i. **d**, Gating strategy to determine the percentage of ALDEFLUOR-positive cells by ALDEFLUOR assay presented on Fig. 2m. **e**, Gating strategy to determine the percentage of $CD24^{-}/CD44^{+}$ cells in ZR-75-1 stable clones presented on Fig. 6b. **f**, Gating strategy to determine the percentage of ALDEFLUOR-positive cells by ALDEFLUOR assay presented on Fig. 6d. **g**, Gating strategy to determine the percentage of $CD24^{-}/CD44^{+}$ cells in primary spheres derived from the indicated MCF7 stable clones presented on Fig. 6h. **h**, Gating strategy to determine the percentage of $CD24^{-}/CD44^{+}$ cells in BT-474 stable clones presented on Supplementary Fig. 11f . Frequencies of cell population after gating are indicated in the gates.

Supplementary Table 1. A list of oligonucleotide primers used in the study

Name	Sequence
For human RNase 1 cloning in pCDH-CMV-MCS-EF1 expression plasmid	
pCDH-R1_F	ACGCGAATTCGCCACCATGGCTCTGGA
pCDH-R1_R	ACGCGGATCCTCACTTGTCGTCATCGTCTTTGTAG TCGGCGGTAGAGTCCTCCACAGA
For human RNase 1 mutagenesis	
R1-H12A_F	GGCAGGCTATGGACTCAGACAGTTCCC
R1-H12A_R	AGTCCATAGCCTGCCGCTGGAATTTCTTG
For shRNA targeting human RNase 1	
sh-R1#1 (V3LHS_313141)	TACAGTAGGTGGAGCTGCT
sh-R1#2 (V2LHS_32407)	TTGGTTACAGTAGGTGGAG
For shRNA targeting human EphA4	
sh-A4#1 (TRCN0000344511)	CCGGGACTTGCAAGGAGACGTTTAACTCGAGTTA AACGTCTCCTTGCAAGTCTTTTG
sh-A4#2 (TRCN0000196950)	CCGGGACTTGCAAGGAGACGTTTAACTCGAGTTA AACGTCTCCTTGCAAGTCTTTTTG
For knockout targeting human RNase 1 (KO-R1)	
hRNase 1-1	TATTCCGGCGCCTCATCATT (271 to 293, – strand)
hRNase 1-2	CACCTTTGTGCACGAGCCCC (322 to 344, + strand)
hRNase 1-3	GTCTCTCCTTCGGGCTGGTC (481 to 503, – strand)
For knockout targeting human EphA4 (KO-A4)	
EphA4-1	TGTGGGAAGTGATGTCGTAC (2551 to 2573, + strand)
EphA4-2	GGCACCGGCGAACCATGGCT (97 to 119, + strand)
EphA4-3	CGACGCTGTCACAGGTTCCA (161 to 183, + strand)

Supplementary Table 2. A list of primary antibodies used in the study

Antibody	Supplier	Catalog No.	Appl.[a]	Usage
Anti-Human β-Actin, clone AC-74	Sigma-Aldrich	#A2228	WB	1:10,000
Anti-Human Akt	Cell Signaling Technology	#9272	WB	1:1,000
Anti-Human Phospho-Akt (Ser473)	Cell Signaling Technology	#9271	WB	1:1,000
PE-Cy™7 Mouse Anti-Human CD24, Clone ML5	BD Biosciences	#561646	FC	1:100
APC Mouse Anti-Human CD44, Clone G44-26	BD Biosciences	#559942	FC	1:60
Anti-Human CD133 [EPR20980-104]	Abcam	#ab216323	IHC	1:200
Anti-Human Erk1/2	EMD Millipore	#06-182	WB	1:10,000
Anti-Human Phospho-Erk1/2 (Thr202/Tyr204) (D13.14.4E)	Cell Signaling Technology	#4370	WB	1:10,000
Anti-Human EphA3 (D-2)	Santa Cruz Biotechnology	#sc-514209	WB	1:500
Anti-Human EphA4 (N-terminal region), Clone M280	ECM Biosciences	#EM2801	WB	1:1,000
Anti-Human EphA4 (S-20)	Santa Cruz Biotechnology	#sc-921	PLA /IP	1:50 /4 μg
Anti-Human EphA4 (6H7)	EMD Millipore	#AP1173	ELISA	3 μg/ml
Anti-Human phospho-EphA4 (Y602)	ECM Biosciences	#EP2731	WB	1:1,000
Anti-Human phospho-EphA4 (Y779)	ECM Biosciences	#EP2751	WB	1:1,000
Anti-Human phospho-EphA4 (Y779/833)	LifeSpan BioSciences	#LS-C381624	IHC	1:150
Anti-Human EphA5 (L-15)	Santa Cruz Biotechnology	#sc-1014	WB /IP	1:500 /6 μg
Anti-Human Ephrin-A1 (40-120 aa, Internal)	LifeSpan BioSciences	#LS-C383378	WB	1:1,000
Anti-Human Ephrin-A2 (C-Terminus)	LifeSpan BioSciences	#LS-C352139	WB	1:1,000
Anti-Human Ephrin-A3 [N1C3]	GeneTex	#GTX101455	WB	1:500
Anti-Human Ephrin-A4	R & D Systems	#AF369	WB	1:1,000
Anti-Human Ephrin-A5	R & D Systems	#AF3743	WB	1:1,000

Anti-Human Ephrin-A5 (Biotin, aa21-203)	LifeSpan BioSciences	#LS-C688235	ELISA	1:500
Anti-Human Ephrin-B1	Proteintech	#12999-1-AP	WB	1:1,000
Anti-Human Ephrin-B2 [N1C1]	GeneTex	#GTX105582	WB	1:1,000
Anti-Human Ephrin-B3	R & D Systems	#MAB395	WB	1:500
Monoclonal ANTI-FLAG® M2 antibody produced in mouse	Sigma-Aldrich	#F3165	WB /PLA	1:3,000 /1:100
Anti-GST (91G1)	Cell Signaling Technology	#2625	WB	1:3,000
Anti-Myc Tag, clone 4A6	EMD Millipore	#05-724	WB	1:3,000
Anti-Human p50 (E-10)	Santa Cruz Biotechnology	#sc-8414	WB	1:1,000
Anti-Human p65 (D14E12)	Cell Signaling Technology	#8242	WB	1:1,000
Anti-Human Phospho-p65 (S536) (93H1)	Cell Signaling Technology	#3033	WB	1:1,000
Anti-Human PLCγ1	Cell Signaling Technology	#2822	WB	1:1,000
Anti-Human Phospho-PLCγ1 (Y783)	Sigma-Aldrich	#SAB4503827	WB	1:1,000
Anti-Human RNase1	Sigma-Aldrich	#HPA001140	WB /IHC	1:1,000 /1:100
Anti-Human RNase1 (Biotin, aa29-156)	LifeSpan BioSciences	#LS-C299825	ELISA	1:500
Anti-Mouse RNase 1 (T-12)	Santa Cruz Biotechnology	#sc-169198	WB	1:1,000
Anti-Human Src (36D10)	Cell Signaling Technology	#2109	WB	1:1,000
Anti-Human Phospho-Src Family (Tyr416) (D49G4)	Cell Signaling Technology	#6943	WB	1:1,000
Anti-Human Tubulin, clone B-5-1-2	Sigma-Aldrich	#T5168	WB	1:10,000

[a]ELISA, Enzyme-Linked Immunosorbent Assay; FC, Flow Cytometry; IHC, Immunohistochemistry; IP, Immunoprecipitation; PLA, Proximity Ligation Assay; WB, Western Blotting.
