# Supplementary material for: Human ribonuclease 1 serves as a secretory ligand of ephrin A4 receptor and induces breast tumor initiation
Source: Nat Commun. 2021 May 13;12:2788. doi: 10.1038/s41467-021-23075-2 (PMC8119676; doi:10.1038/s41467-021-23075-2)
Supplement: Supplementary file 3 — Reporting summary. [file 41467_2021_23075_MOESM3_ESM.pdf]

natureresearch

Corresponding author(s): Dr. Mien-Chie Hung

# Reporting Summary

Nature Research wishes to improve the reproducibility of the work that we publish. This form provides structure for consistency and transparency in reporting. For further information on Nature Research policies, see Authors & Referees and the Editorial Policy Checklist.

## Statistical parameters

When statistical analyses are reported, confirm that the following items are present in the relevant location (e.g. figure legend, table legend, main text, or Methods section).

| n/a | Confirmed | |
|---|---|---|
| ☐ | ☒ | The exact sample size (*n*) for each experimental group/condition, given as a discrete number and unit of measurement |
| ☐ | ☒ | An indication of whether measurements were taken from distinct samples or whether the same sample was measured repeatedly |
| ☐ | ☒ | The statistical test(s) used AND whether they are one- or two-sided<br>*Only common tests should be described solely by name; describe more complex techniques in the Methods section.* |
| ☒ | ☐ | A description of all covariates tested |
| ☒ | ☐ | A description of any assumptions or corrections, such as tests of normality and adjustment for multiple comparisons |
| ☐ | ☒ | A full description of the statistics including central tendency (e.g. means) or other basic estimates (e.g. regression coefficient) AND variation (e.g. standard deviation) or associated estimates of uncertainty (e.g. confidence intervals) |
| ☐ | ☒ | For null hypothesis testing, the test statistic (e.g. *F*, *t*, *r*) with confidence intervals, effect sizes, degrees of freedom and *P* value noted<br>*Give P values as exact values whenever suitable.* |
| ☒ | ☐ | For Bayesian analysis, information on the choice of priors and Markov chain Monte Carlo settings |
| ☒ | ☐ | For hierarchical and complex designs, identification of the appropriate level for tests and full reporting of outcomes |
| ☐ | ☒ | Estimates of effect sizes (e.g. Cohen's *d*, Pearson's *r*), indicating how they were calculated |
| ☐ | ☒ | Clearly defined error bars<br>*State explicitly what error bars represent (e.g. SD, SE, CI)* |

*Our web collection on statistics for biologists may be useful.*

## Software and code

Policy information about availability of computer code

| | |
|---|---|
| Data collection | Western blotting data were collected using ImageQuant LAS 4000 (GE Healthcare Life Sciences). Flow cytometry data were acquired using BD FACSDiva 8.0.2 (BD Biosciences). |
| Data analysis | GraphPad Prism (version 8.0.0; GraphPad), ImageJ software program (version 1.52a; NIH), FlowJo software (version 10.7.1; BD Biosciences) |

For manuscripts utilizing custom algorithms or software that are central to the research but not yet described in published literature, software must be made available to editors/reviewers upon request. We strongly encourage code deposition in a community repository (e.g. GitHub). See the Nature Research guidelines for submitting code & software for further information.

## Data

Policy information about availability of data

All manuscripts must include a data availability statement. This statement should provide the following information, where applicable:

- Accession codes, unique identifiers, or web links for publicly available datasets
- A list of figures that have associated raw data
- A description of any restrictions on data availability

The publicly available databases used in this study are available in the Kaplan-Meier plotter database (http://kmplot.com/analysis/), the Gene Expression patterns

across Normal and Tumor tissues named GENT2 (http://gent2.appex.kr/gent2/), and the UCSC Cancer Genome Browser (http://xena.ucsc.edu/welcome-to-ucsc-xena/). Source data are provided with this paper. The remaining data are available within the Article, Supplementary Information or available from the authors upon request.

# Field-specific reporting

Please select the best fit for your research. If you are not sure, read the appropriate sections before making your selection.

☒ Life sciences ☐ Behavioural & social sciences ☐ Ecological, evolutionary & environmental sciences

For a reference copy of the document with all sections, see nature.com/authors/policies/ReportingSummary-flat.pdf

# Life sciences study design

All studies must disclose on these points even when the disclosure is negative.

| | |
|---|---|
| Sample size | Sample sizes for in vitro and in vivo experiments were determined on the basis of prior knowledge of variation, including papers with similar concept of RNase family in human cancers (Wang et al. Cancer Cell. 2018 PMID: 29606349; Liu et al. J Hepatol. 2021 PMID: 33031845). No statistical method was used to predetermine sample size as sample size selection with the above published methods is sufficient to detect meaningful biological differences with good reproducibility. |
| Data exclusions | No data were excluded from the analyses. |
| Replication | Our experimental findings were confirmed with at least two times independent experiments. |
| Randomization | For in vitro experiments, cells were randomly allocated into control and experimental groups. For in vivo experiments, age and sex-matched mice were randomized into control and experimental groups prior to tumor size measurement and inhibitor treatment. Randomization was not relevant to the experiments using clinical cohorts. Samples of noncancerous individuals and cancer patients were collected and analyzed identically regardless of other clinical history or external criteria. |
| Blinding | IHC experiment was performed by the pathologists without any information about patient tissue. ELISA experiment was performed without any information about patient serum. Blinding was not used for animal works because the investigators needed to know the treatment groups in order to perform experiments. Blinding was not applicable to the rest of other in vitro experiments (e.g. Western blotting, sphere-forming assay, flow cytometric analysis) because the same investigator was doing group allocation during data collection and/or analysis. |

# Behavioural & social sciences study design

All studies must disclose on these points even when the disclosure is negative.

| | |
|---|---|
| Study description | *Briefly describe the study type including whether data are quantitative, qualitative, or mixed-methods (e.g. qualitative cross-sectional, quantitative experimental, mixed-methods case study).* |
| Research sample | *State the research sample (e.g. Harvard university undergraduates, villagers in rural India) and provide relevant demographic information (e.g. age, sex) and indicate whether the sample is representative. Provide a rationale for the study sample chosen. For studies involving existing datasets, please describe the dataset and source.* |
| Sampling strategy | *Describe the sampling procedure (e.g. random, snowball, stratified, convenience). Describe the statistical methods that were used to predetermine sample size OR if no sample-size calculation was performed, describe how sample sizes were chosen and provide a rationale for why these sample sizes are sufficient. For qualitative data, please indicate whether data saturation was considered, and what criteria were used to decide that no further sampling was needed.* |
| Data collection | *Provide details about the data collection procedure, including the instruments or devices used to record the data (e.g. pen and paper, computer, eye tracker, video or audio equipment) whether anyone was present besides the participant(s) and the researcher, and whether the researcher was blind to experimental condition and/or the study hypothesis during data collection.* |
| Timing | *Indicate the start and stop dates of data collection. If there is a gap between collection periods, state the dates for each sample cohort.* |
| Data exclusions | *If no data were excluded from the analyses, state so OR if data were excluded, provide the exact number of exclusions and the rationale behind them, indicating whether exclusion criteria were pre-established.* |
| Non-participation | *State how many participants dropped out/declined participation and the reason(s) given OR provide response rate OR state that no participants dropped out/declined participation.* |
| Randomization | *If participants were not allocated into experimental groups, state so OR describe how participants were allocated to groups, and if allocation was not random, describe how covariates were controlled.* |

# Ecological, evolutionary & environmental sciences study design

All studies must disclose on these points even when the disclosure is negative.

| | |
|---|---|
| Study description | *Briefly describe the study. For quantitative data include treatment factors and interactions, design structure (e.g. factorial, nested, hierarchical), nature and number of experimental units and replicates.* |
| Research sample | *Describe the research sample (e.g. a group of tagged Passer domesticus, all Stenocereus thurberi within Organ Pipe Cactus National Monument), and provide a rationale for the sample choice. When relevant, describe the organism taxa, source, sex, age range and any manipulations. State what population the sample is meant to represent when applicable. For studies involving existing datasets, describe the data and its source.* |
| Sampling strategy | *Note the sampling procedure. Describe the statistical methods that were used to predetermine sample size OR if no sample-size calculation was performed, describe how sample sizes were chosen and provide a rationale for why these sample sizes are sufficient.* |
| Data collection | *Describe the data collection procedure, including who recorded the data and how.* |
| Timing and spatial scale | *Indicate the start and stop dates of data collection, noting the frequency and periodicity of sampling and providing a rationale for these choices. If there is a gap between collection periods, state the dates for each sample cohort. Specify the spatial scale from which the data are taken* |
| Data exclusions | *If no data were excluded from the analyses, state so OR if data were excluded, describe the exclusions and the rationale behind them, indicating whether exclusion criteria were pre-established.* |
| Reproducibility | *Describe the measures taken to verify the reproducibility of experimental findings. For each experiment, note whether any attempts to repeat the experiment failed OR state that all attempts to repeat the experiment were successful.* |
| Randomization | *Describe how samples/organisms/participants were allocated into groups. If allocation was not random, describe how covariates were controlled. If this is not relevant to your study, explain why.* |
| Blinding | *Describe the extent of blinding used during data acquisition and analysis. If blinding was not possible, describe why OR explain why blinding was not relevant to your study.* |

Did the study involve field work? ☐ Yes ☐ No

## Field work, collection and transport

| | |
|---|---|
| Field conditions | *Describe the study conditions for field work, providing relevant parameters (e.g. temperature, rainfall).* |
| Location | *State the location of the sampling or experiment, providing relevant parameters (e.g. latitude and longitude, elevation, water depth).* |
| Access and import/export | *Describe the efforts you have made to access habitats and to collect and import/export your samples in a responsible manner and in compliance with local, national and international laws, noting any permits that were obtained (give the name of the issuing authority, the date of issue, and any identifying information).* |
| Disturbance | *Describe any disturbance caused by the study and how it was minimized.* |

# Reporting for specific materials, systems and methods

### Materials & experimental systems

| n/a | Involved in the study | |
|---|---|---|
| ☒ | ☐ | Unique biological materials |
| ☐ | ☒ | Antibodies |
| ☐ | ☒ | Eukaryotic cell lines |
| ☒ | ☐ | Palaeontology |
| ☐ | ☒ | Animals and other organisms |
| ☐ | ☒ | Human research participants |

### Methods

| n/a | Involved in the study | |
|---|---|---|
| ☒ | ☐ | ChIP-seq |
| ☐ | ☒ | Flow cytometry |
| ☒ | ☐ | MRI-based neuroimaging |

## Antibodies

Antibodies used

Primary antibodies used in the study are described in the Supplementary Table 2. For example:
Anti-Human RNase1 Antibody, Sigma-Aldrich, Cat#HPA001140, Lot# A78395
PE-Cy™7 Mouse Anti-Human CD24, Clone ML5, BD Biosciences, Cat# 561646, Lot# 9149858
APC Mouse Anti-Human CD44, Clone G44-26, BD Biosciences, Cat# 559942, Lot# 8351667

Validation

All primary antibodies are commercially available and validated by manufacturers.
Anti-Human β-Actin Antibody, Clone AC-74, Sigma-Aldrich, #A2228, Western Blotting, https://www.sigmaaldrich.com/catalog/product/sigma/a2228?lang=en&region=US
Anti-Human Akt Antibody, Cell Signaling Technology, #9272, Western Blotting, https://www.cellsignal.com/products/primary-antibodies/akt-antibody/9272
Anti-Human Phospho-Akt (Ser473) Antibody, Cell Signaling Technology, #9271, Western Blotting, https://www.cellsignal.com/products/primary-antibodies/phospho-akt-ser473-antibody/9271
PE-Cy™7 Mouse Anti-Human CD24, Clone ML5, BD Biosciences, #561646, Flow Cytometry, https://www.bdbiosciences.com/us/applications/research/stem-cell-research/cancer-research/human/pe-cy7-mouse-anti-human-cd24-ml5/p/561646
APC Mouse Anti-Human CD44, Clone G44-26, BD Biosciences, #559942, Flow Cytometry, https://www.bdbiosciences.com/us/applications/research/t-cell-immunology/t-follicular-helper-tfh-cells/surface-markers/human/apc-mouse-anti-human-cd44-g44-26-also-known-as-c26/p/559942
Anti-Human CD133 Antibody [EPR20980-104], Abcam, #ab216323, Immunohistochemistry, https://www.abcam.com/cd133-antibody-epr20980-104-ab216323.html
Anti-Human Erk1/2 Antibody, EMD Millipore, #06-182, Western Blotting, https://www.labome.com/product/EMD-Millipore/06-182.html
Anti-Human Phospho-Erk1/2 (Thr202/Tyr204) (D13.14.4E), Cell Signaling Technology, #4370, Western Blotting, https://www.cellsignal.com/products/primary-antibodies/phospho-p44-42-mapk-erk1-2-thr202-tyr204-d13-14-4e-xp-rabbit-mab/4370
Anti-Human EphA3 Antibody (D-2), Santa Cruz Biotechnology, #sc-514209, Western Blotting, https://www.scbt.com/p/epha3-antibody-d-2?requestFrom=search
Anti-Human EphA4 (N-terminal region) Antibody, Clone M280, ECM Biosciences, #EM2801, Western Blotting, https://ecmbio.com/products/em2801
Anti-Human EphA4 (S-20) Antibody, Santa Cruz Biotechnology, #sc-921, Proximity Ligation Assay & Immunoprecipitation, https://www.scbt.com/p/epha4-antibody-s-20?requestFrom=search
Anti-Human EPHA4 (6H7) Antibody, EMD Millipore, #AP1173, ELISA, https://www.emdmillipore.com/US/en/product/Anti-EPHA4-Mouse-mAb-6H7,EMD_BIO-AP1173
Anti-Human phospho-EphA4 (Y602) Antibody, ECM Biosciences, #EP2731, Western Blotting, https://ecmbio.com/products/ep2731
Anti-Human phospho-EphA4 (Y779) Antibody, ECM Biosciences, #EP2751, Western Blotting, https://ecmbio.com/products/ep2751
Anti-Human phospho-EphA4 (Y779/833) Antibody, LifeSpan BioSciences, #LS-C381624, Immunohistochemistry, https://www.lsbio.com/antibodies/epha3-epha4-epha5-antibody-phospho-tyr779-833-elisa-ihc-ls-c381624/393725
Anti-Human EphA5 (L-15) Antibody, Santa Cruz Biotechnology, #sc-1014, Western Blotting & Immunoprecipitation, https://www.scbt.com/p/epha5-antibody-l-15?requestFrom=search
Anti-Human Ephrin-A1 (40-120 aa, Internal) Antibody, LifeSpan BioSciences, #LS-C383378, Western Blotting, https://www.lsbio.com/antibodies/efna1-antibody-ephrin-a1-antibody-40-120-aa-internal-elisa-if-immunofluorescence-ihc-wb-western-ls-c383378/395479
Anti-Human Ephrin-A2 (C-Terminus) Antibody, LifeSpan BioSciences, #LS-C352139, Western Blotting, https://www.lsbio.com/antibodies/efna2-antibody-ephrin-a2-antibody-c-terminus-wb-western-ls-c352139/363260
Anti-Human Ephrin-A3 [N1C3] Antibody, GeneTex, #GTX101455, Western Blotting, https://www.genetex.com/Product/Detail/Ephrin-A3-antibody-N1C3/GTX101455
Anti-Human Ephrin-A4 Antibody, R & D Systems, #AF369, Western Blotting, https://www.rndsystems.com/products/human-ephrin-a4-antibody_af369
Anti-Human Ephrin-A5 Antibody, R & D Systems, #AF3743, Western Blotting, https://www.rndsystems.com/products/human-ephrin-a5-antibody_af3743
Anti-Human Ephrin-A5 Antibody (Biotin, aa21-203), LifeSpan BioSciences, #LS-C688235, ELISA, https://www.lsbio.com/antibodies/efna5-antibody-ephrin-a5-antibody-aa21-203-biotin-wb-western-ls-c688235/713663
Anti-Human Ephrin-B1 Antibody, Proteintech, #12999-1-AP, Western Blotting, https://www.ptglab.com/products/EFNB1-Antibody-12999-1-AP.htm
Anti-Human Ephrin-B2 [N1C1] Antibody, GeneTex, #GTX105582, Western Blotting, https://www.genetex.com/Product/Detail/Ephrin-B2-antibody-N1C1/GTX105582
Anti-Human Ephrin-B3 Antibody, R & D Systems, #MAB395, Western Blotting, https://www.rndsystems.com/products/human-ephrin-b3-antibody-88838_mab395
Monoclonal ANTI-FLAG® M2 antibody produced in mouse, Sigma-Aldrich, #F3165, Western Blotting & Proximity Ligation Assay, https://www.sigmaaldrich.com/catalog/product/sigma/f3165?lang=en&region=US
Anti-GST (91G1) Rabbit mAb, Cell Signaling Technology, #2625, Western Blotting, https://www.cellsignal.com/products/primary-antibodies/gst-91g1-rabbit-mab/2625
Anti-Myc Tag Antibody, clone 4A6, EMD Millipore, #05-724, Western Blotting, https://www.emdmillipore.com/US/en/product/Anti-Myc-Tag-Antibody-clone-4A6,MM_NF-05-724
Anti-Human p50 (E-10) Antibody, Santa Cruz Biotechnology, #sc-8414, Western Blotting, https://www.scbt.com/p/nfkappab-p50-antibody-e-10?requestFrom=search
Anti-Human p65 (D14E12) Antibody, Cell Signaling Technology, #8242, Western Blotting, https://www.cellsignal.com/products/primary-antibodies/nf-kb-p65-d14e12-xp-rabbit-mab/8242
Anti-Human Phospho-p65 (S536) (93H1) Antibody, Cell Signaling Technology, #3033, Western Blotting, https://www.cellsignal.com/products/primary-antibodies/phospho-nf-kb-p65-ser536-93h1-rabbit-mab/3033
Anti-Human PLCγ1 Antibody, Cell Signaling Technology, #2822, Western Blotting, https://www.cellsignal.com/products/primary-

antibodies/plcg1-antibody/2822
Anti-Human Phospho-PLCγ1 (Y783) Antibody, Sigma-Aldrich, #SAB4503827, Western Blotting, https://www.sigmaaldrich.com/catalog/product/sigma/sab4503827?lang=en&region=US
Anti-Human RNase1 Antibody, Sigma-Aldrich, #HPA001140, Western Blotting & Immunohistochemistry, https://www.sigmaaldrich.com/catalog/product/sigma/hpa001140?lang=en&region=US
Anti-Human RNase1 Antibody (Biotin, aa29-156), LifeSpan BioSciences, #LS-C299825, ELISA, https://www.lsbio.com/antibodies/ribonuclease-a-antibody-rnase1-antibody-aa29-156-biotin-wb-western-ls-c299825/309740
Anti-Mouse RNase 1 (T-12) Antibody, Santa Cruz Biotechnology, #sc-169198, Western Blotting, https://datasheets.scbt.com/sc-169198.pdf
Anti-Human Src (36D10) Antibody, Cell Signaling Technology, #2109, Western Blotting, https://www.cellsignal.com/products/primary-antibodies/src-36d10-rabbit-mab/2109
Anti-Human Phospho-Src Family (Tyr416) (D49G4) Antibody, Cell Signaling Technology, #6943, Western Blotting, https://www.cellsignal.com/products/primary-antibodies/phospho-src-family-tyr416-d49g4-rabbit-mab/6943
Anti-Human Tubulin Antibody, clone B-5-1-2, Sigma-Aldrich, #T5168, Western Blotting, https://www.sigmaaldrich.com/catalog/product/sigma/t5168?lang=en&region=US

## Eukaryotic cell lines

Policy information about cell lines

Cell line source(s): American Type Culture Collection (ATCC)

Authentication: Cells were authenticated by short tandem repeat DNA finger printing.

Mycoplasma contamination: They were negative for mycoplasma.

Commonly misidentified lines (See ICLAC register): NO

## Animals and other organisms

Policy information about studies involving animals; ARRIVE guidelines recommended for reporting animal research

Laboratory animals: BALB/c nude female mice of six week of age were purchased from The Jackson Laboratory. Mice were maintained at an ambient temperature of 70 ± 2°F and relative humidity of 30–70% under a 12-h light/12-h dark cycle. All animal procedures were conducted under the approval of the IACUC at MD Anderson.

Wild animals: No wild animals were used in the study.

Field-collected samples: No field-collected samples were used in the study.

## Human research participants

Policy information about studies involving human research participants

Population characteristics: The study included all female patients with breast cancer, regardless of age range or molecular subtypes. We provided clinical information of mean and median ages of each cohort in the Methods section. Age, gender, genotypic background, and therapeutic history were not treated as covariates in this study.

Recruitment: Sample recruitment in this study was from all female patients with breast cancer across multiple institutes following the guidelines approved by the Institutional Review Board at The University of Texas MD Anderson Cancer Center, Harbin Medical University Cancer Hospital, China Medical University Hospital, Taipei Veterans General Hospital, and Kaohsiung Medical University Hospital. Written informed consent was obtained from all patients. For the experiments comparing normal and cancer serum samples, female breast cancer patients were recruited regardless of age range or molecular subtypes. For the experiment using the paired plasma and tissue samples, patients for whom samples were not available of both plasma and tissue were not considered for inclusion in this study. All clinical information validated our results without selection bias.

## Flow Cytometry

### Plots

Confirm that:

☒ The axis labels state the marker and fluorochrome used (e.g. CD4-FITC).

☒ The axis scales are clearly visible. Include numbers along axes only for bottom left plot of group (a 'group' is an analysis of identical markers).

☒ All plots are contour plots with outliers or pseudocolor plots.

☒ A numerical value for number of cells or percentage (with statistics) is provided.

## Methodology

| | |
|---|---|
| Sample preparation | All samples used in this study were breast cancer stable cell lines from cell culture, which were trypsinized and harvested to create cell suspensions in Cell Staining Buffer in triplicates. The collected cells were then stained with PE-Cy™7-conjugated anti-CD24 and APC-conjugated anti-CD44 antibodies for 20 min in dark by using PE-Cy™7 Mouse IgG2a and APC Mouse IgG2b as isotype control staining. We provide the information in the Methods section. |
| Instrument | BD FACSCanto II cytometer |
| Software | BD FACSDiva 8.0.2 software and FlowJo 10.7.1 software |
| Cell population abundance | Cell population data were collected on a debris exclusion gate at the time of acquisition of FACSDiva software. A total of 10,000 cell events per sample was collected. The percentage of cell population after gating are indicated in the Supplementary Figure 15. No cell sorting was performed in this study. |
| Gating strategy | For conventional gating strategy with double-staining, the scatter of FSC-A vs. SSC-A was performed. Remaining gates were drawn based on cell populations between negative (isotype controls) and positive (single-staining) staining to serve as a basis for comparison with the double-staining data. Gating strategy with the percentage of the starting cell population determined on FSC-A/SSC-A is exemplified in the Supplementary Figure 15. |

☒ Tick this box to confirm that a figure exemplifying the gating strategy is provided in the Supplementary Information.
